# High-risk pulmonary embolism in a post-COVID 19 female under hormonal contraception

**DOI:** 10.1093/ehjcr/ytad547

**Published:** 2023-11-06

**Authors:** Andrea Sonaglioni, Michele Lombardo, Adriana Albini, Sergio Harari

**Affiliations:** Department of Cardiology, Istituto di Ricovero e Cura a Carattere Scientifico (IRCCS) MultiMedica, Via San Vittore 12, 20123 Milan, Italy; Department of Cardiology, Istituto di Ricovero e Cura a Carattere Scientifico (IRCCS) MultiMedica, Via San Vittore 12, 20123 Milan, Italy; Scientific Directorate, European Institute of Oncology (IEO) Istituto di Ricovero e Cura a Carattere Scientifico (IRCCS), Milan, Italy; Department of Clinical Sciences and Community Health, University of Milan, Milan, Italy

A 29-year-old, nulliparous, non-smoker, overweight (body surface area 1.97 m^2^; body mass index 29.4 kg/m^2^) female presented to the emergency room of our hospital for ongoing dyspnoea, tachycardia, and general malaise. She had a recent COVID-19 infection, presenting with mild symptoms (pharyngodynia only), which occurred 4 weeks before and lasted 1 week. Moreover, she had a history of chronic oral contraceptive pill (OCP, levonorgestrel 0.10 mg and ethinylestradiol 0.02 mg daily) use for 6 years. At hospital admission, blood pressure was 90/60 mmHg, heart rate was 132 b.p.m., arterial oxygen saturation was 88%, and body temperature was 36.5°C. Arterial blood gas analysis showed hypocapnia (pCO_2_ 27.6 mmHg) and hypoxaemia (pO_2_ 66 mmHg) with mild respiratory alkalosis (pH 7.48). The electrocardiogram revealed sinus rhythm with an S1Q3T3 pattern (*Figure 1A*). A bedside transthoracic echocardiography (TTE) showed saddle pulmonary embolism (PE, *Figure 1B*), with McConnell’s sign (right ventricular free wall akinesis with sparing of the apex) due to severe pulmonary hypertension (tricuspid regurgitation velocity >3.4 m/s). An urgent computed tomography (CT) pulmonary angiography confirmed extensive saddle PE (*Figure 1C*). Computed tomography venography documented concurrent thrombosis of the left saphenofemoral junction and great saphenous vein (*Figure 1D*). The estimated Pulmonary Embolism Severity Index score was 119 (Class IV, high 30-day mortality risk, 4.0–11.4%). Accordingly, the patient underwent systemic thrombolysis with a recombinant tissue plasminogen activator (rtPA) 100 mg infusion over 2 h, followed by enoxaparin sodium 6000 IU subcutaneously twice daily. After treatment, she showed a rapid improvement in her haemodynamic and ventilatory patterns, the electrocardiogram quickly normalized, and saddle PE disappeared on TTE (*Figure 1E*). The further use of OCP was contraindicated, and, on Day 6 after presentation, the patient was discharged from our institution with the indication of 6 months of anticoagulant therapy with apixaban 5 mg twice a day.

**Figure 1:**
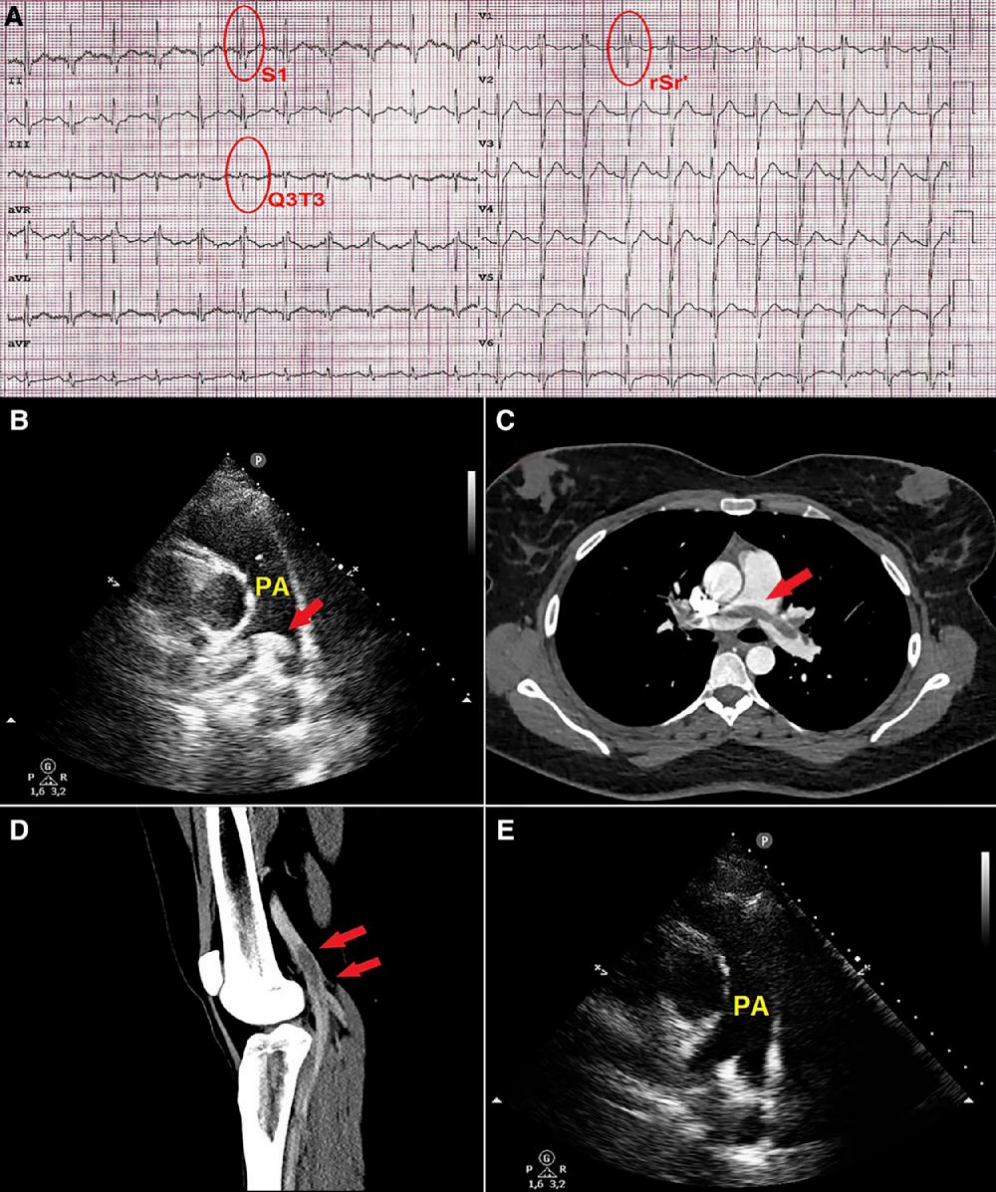
(*A*) Twelve-lead electrocardiogram, showing mild right ventricular delay and S1Q3T3 pattern, indicative of acute pulmonary embolism. (*B*) 2D transthoracic echocardiography. Basal short-axis view, demonstrating a saddle pulmonary embolism (arrow) extending in both the right and left pulmonary arteries. PA, pulmonary artery. (*C*) Axial view of the computed tomographic pulmonary angiography, showing a saddle pulmonary embolism (arrow) extending in both the right and left pulmonary arteries. (*D*) Computed tomography venography in sagittal reconstruction, showing concurrent thrombosis of the left saphenofemoral junction and great saphenous vein (arrows). (*E*) Basal short-axis view from transthoracic echocardiography, demonstrating total regression of mobile saddle thrombus after thrombolytic treatment. PA, pulmonary artery.

To date, only two authors have described the association between chronic OCP use and venous thromboembolism/PE in the acute phase of COVID-19 disease.^1,2^ Differently from these authors who reported segmental or subsegmental cases of PE in acute COVID-19 patients, we described a case of proximal PE detected during the post-acute phase of COVID-19 disease. Given that during the convalescent phase of COVID-19 disease, a state of endothelial dysfunction, hypercoagulability, and low-grade inflammation may be persistent,^3,4^ it is likely that OCP may have exerted an additive prothrombotic effect on a substrate of chronic immuno-thrombogenicity related to COVID-19 infection.

## Data Availability

The data underlying this article will be shared upon reasonable request to the corresponding author.
